# ColocQuiaL: a QTL-GWAS colocalization pipeline

**DOI:** 10.1093/bioinformatics/btac512

**Published:** 2022-07-27

**Authors:** Brian Y Chen, William P Bone, Kim Lorenz, Michael Levin, Marylyn D Ritchie, Benjamin F Voight

**Affiliations:** School of Arts and Sciences, University of Pennsylvania, Philadelphia, PA 19104, USA; Genomics and Computational Biology Graduate Group, Perelman School of Medicine, University of Pennsylvania, Philadelphia, PA 19104, USA; Department of Systems Pharmacology and Translational Therapeutics, Perelman School of Medicine, University of Pennsylvania, Philadelphia, PA 19104, USA; Department of Genetics, University of Pennsylvania, Philadelphia, PA 19104, USA; Corporal Michael J. Crescenz VA Medical Center, Philadelphia, PA 19104, USA; Corporal Michael J. Crescenz VA Medical Center, Philadelphia, PA 19104, USA; Department of Surgery, Perelman School of Medicine, University of Pennsylvania, Philadelphia, PA 19104, USA; Division of Cardiovascular Medicine, Department of Medicine, University of Pennsylvania Perelman School of Medicine, Philadelphia, PA 19104, USA; Department of Genetics, University of Pennsylvania, Philadelphia, PA 19104, USA; Institute for Biomedical Informatics, Perelman School of Medicine, University of Pennsylvania, Philadelphia, PA 19104, USA; Center for Precision Medicine, Perelman School of Medicine, University of Pennsylvania, Philadelphia, PA 19104, USA; Department of Systems Pharmacology and Translational Therapeutics, Perelman School of Medicine, University of Pennsylvania, Philadelphia, PA 19104, USA; Department of Genetics, University of Pennsylvania, Philadelphia, PA 19104, USA; Corporal Michael J. Crescenz VA Medical Center, Philadelphia, PA 19104, USA; Institute for Biomedical Informatics, Perelman School of Medicine, University of Pennsylvania, Philadelphia, PA 19104, USA; Institute for Translational Medicine and Therapeutics, Perelman School of Medicine, University of Pennsylvania, Philadelphia, PA 19104, USA

## Abstract

**Summary:**

Identifying genomic features responsible for genome-wide association study (GWAS) signals has proven to be a difficult challenge; many researchers have turned to colocalization analysis of GWAS signals with expression quantitative trait loci (eQTL) and splicing quantitative trait loci (sQTL) to connect GWAS signals to candidate causal genes. The ColocQuiaL pipeline provides a framework to perform these colocalization analyses at scale across the genome and returns summary files and locus visualization plots to allow for detailed review of the results. As an example, we used ColocQuiaL to perform colocalization between a recent type 2 diabetes GWAS and Genotype-Tissue Expression (GTEx) v8 single-tissue eQTL and sQTL data.

**Availability and implementation:**

ColocQuiaL is primarily written in R and is freely available on GitHub: https://github.com/bvoightlab/ColocQuiaL.

## 1 Introduction

Genome-wide association studies (GWAS) conducted on large populations have identified a plethora of associations between genetic variation and complex traits and diseases in humans (Buniello *et al.*, 2019). From this collection of predominantly non-coding variants, a central challenge has emerged to identify which genomic features at each locus ultimately play a functional role in the phenotype of interest. This insight is a key barrier to initiate functional follow-up experiments. One source of data that can be used to link GWAS associations to a predicted effector transcript of action is by connecting them with molecular phenotype quantitative trait loci (QTLs). A well-powered source of two important types of QTLs—those associated with variation in expression of transcripts (eQTLs) and proportion of alternatively spliced transcripts (sQTLs)—was reported across >40 tissues by the Genotype-Tissue Expression (GTEx) project (Carithers *et al.*, 2015). To connect trait signals to these data and identify potential candidate genes, the community has turned to statistical colocalization—an approach designed to infer if the association signals between a complex trait and QTL are tagged by the same genetic variant(s) (Giambartolomei *et al.*, 2014). To provide a common, reproducible framework to perform colocalization analyses between QTL and complex trait data at moderate computational scale, we present here an implementation, ColocQuiaL, which allows for the rapid execution of colocalization analyses for GWAS signals from a summary statistics file with QTL signals from the eQTL or sQTL datasets of a user’s choosing. As a proof of concept, we applied it to a large catalog of lead associations and summary data for type 2 diabetes (T2D) and the GTEx v8 single-tissue eQTLs or sQTLs datasets (Mahajan *et al.*, 2020; Vujkovic *et al.*, 2020).

## 2 ColocQuiaL

**Fig. 1.**
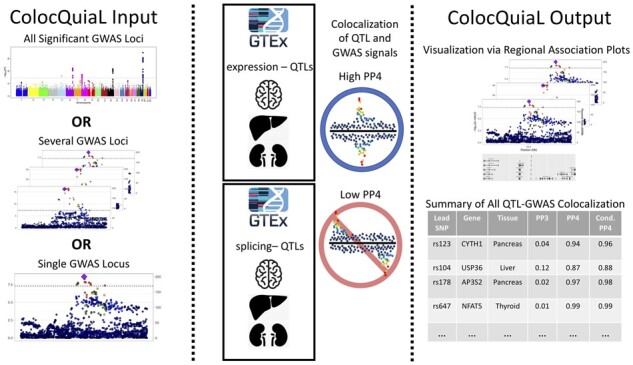
ColocQuiaL workflow. The first panel shows the possible GWAS inputs that ColocQuiaL accepts. The second panel demonstrates how ColocQuiaL performs colocalizations between the available QTL signals and the GWAS signals provided. The last panel demonstrates the regional association plots and the summary of colocalization results output that ColocQuiaL provides

The motivation underlying the development of ColocQuiaL was the need to perform and visualize the results from a large number (10 000+) of colocalization analyses between signals for one (or more) complex traits and the catalog of available human tissue QTL data (Fig. 1). As such, ColocQuiaL automates the execution of COLOC to perform colocalization analyses between GWAS signals for any trait of interest and single-tissue eQTL and sQTL signals (Giambartolomei *et al.*, 2014). The input loci to ColocQuiaL can be a single GWAS locus, a list of GWAS loci of interest, or the summary statistics across the entire genome (Fig. 1). Users can specify the lead SNPs and the genomic intervals of the colocalization analysis based on prior knowledge of the loci, or they can perform more general analyses by supplying the GWAS summary statistics file and their preferred definition of significant *P*-values and independent loci via an interface with PLINK (Purcell *et al.*, 2007). In all these scenarios, ColocQuiaL will perform a colocalization analysis between each single-tissue eQTL or sQTL signal for which a lead SNP is a significant QTL and the GWAS signal at the locus. ColocQuiaL generates output files to allow for both manual review of individual colocalization analyses and quick review of all the analyses performed (Fig. 1).

The majority of these output files are deposited in lead SNP specific directories. The COLOC results and intermediary files for each colocalization analysis at a lead SNP will all be saved to the directory specific to the lead SNP. This directory will also include regional association plots for each QTL-tissue signal involved in a colocalization analysis and the GWAS trait signal at the locus. These regional association plots are similar to those generated by the popular tool LocusZoom, but are generated as part of the ColocQuiaL code (Fig. 1) (Pruim *et al.*, 2011). Finally, ColocQuiaL generates a summary output file that contains all of the locus level posterior probabilities for the COLOC analyses of the ColocQuiaL run (Fig. 1). The ColocQuiaL pipeline is written in R (v3.6.3 or later) and bash. It executes COLOC with its default priors and is compatible with at least COLOC versions 4 and 5. We implemented a version of ColocQuiaL that is parallelized at the lead SNP level via the LSF workload submission system and an in-series version that can be modified for other job submission systems. ColocQuiaL also interfaces with the following standard bioinformatic tools: PLINK (v 1.90Beta45), bedtools (v2.29.1) and Tabix (0.2.5) (Li, 2011; Purcell *et al.*, 2007; Quinlan and Hall, 2010). In order to run the pipeline, the user will need to configure a small number of dependency files from the summary statistics of the QTL dataset they wish to use for colocalization analyses. Detailed instructions on how to download and configure the dependency files for GTEx v8 single-tissue files from the GTEx Portal as well as eQTL Catalogue data from their website are available at https://github.com/bvoightlab/ColocQuiaL. These procedures should also apply to any other eQTL or sQTL dataset for which summary statistics are available.

## 3 Usage scenario

As a use case, we used ColocQuiaL to perform colocalization analysis of all reported independent T2D genome-wide significant signals reported in Mahajan *et al.* (2020) with GTEx single-tissue eQTLs and sQTLs using the Vujkovic *et al.* (2020) T2D summary statistics (Mahajan *et al.*, 2020; Vujkovic *et al.*, 2020). We used the list of 520 genome-wide significant (*P*-value ≤ 5 × 10^−8^) lead SNPs reported in Mahajan *et al.* (2020) as the GWAS loci input for ColocQuiaL, and used the GTEx v8 significant eQTL/sQTL files as the reference for significant QTLs. For this analysis, we considered a conditional posterior probability of colocalization of 0.8 or greater to be evidence of colocalization between the T2D signal and the QTL signal. The conditional posterior probability of colocalization is the posterior probability of there being two significant signals at a locus that colocalize (PP4) divided by the sum of the PP4 and the posterior probability that there are two significant signals at the locus that do not colocalize (PP3). We chose to use this metric to assess colocalization since all GWAS and QTL signals in this analysis have been defined as significant in the Mahajan *et al.* (2020) or GTEx analyses and the posterior probability of the other COLOC hypotheses should be negligible. Across the 520 T2D lead SNPs, we found 278 colocalized (PP4/(PP3 + PP4) ≥ 0.8) with one or more eQTL signals and 148 colocalized with one or more sQTL signals. These colocalizing signals represent 766 genes and 47 tissues among the eQTLs and 268 genes and 48 tissues among the sQTLs. In total, we performed 9563 colocalizations between T2D signals and eQTL signals and 38 994 between T2D signals and sQTL signals. We performed this on a PowerEdge R630 Server (2.2Ghz Xeon E5-2699 v4 Dual 22-Core, 512 Gb memory) using the lead SNP parallelized version of ColocQuiaL. The median run time and median maximum memory usage for each lead SNP job were 10 min 1 s and 17.66 GBs for the eQTLs and 7 min 49 s and 16.56 GBs for sQTLs. Both eQTLs and sQTLs had a small number of outlier lead SNPs that were significant for a much larger number of eQTL/sQTL signals in GTEx than the average lead SNP, with the maximum number of colocalizations required being 343 for an eQTL lead SNP and 2561 for an sQTL lead SNP. Our results show these T2D GWAS signals colocalize with QTL signals for many of the genes one would expect and replicate recent T2D colocalization studies. We found three maturity-onset diabetes of the young (MODY) gene QTLs colocalized with T2D signals. One MODY gene, *KCNJ11*, had both an eQTL and an sQTL signal that colocalized with T2D signals (Naylor *et al.*, 2018). We also compared our findings to a predicted causal genes list for T2D (from the T2D knowledge portal) and found that T2D signals colocalized with eQTL or sQTL signals for 22 out of the 58 genes. Finally, we compared our results to the recently published T2D QTL colocalization result from Gloudemans *et al.* (2022)—colocalization of T2D and insulin resistance GWAS data with eQTLs and sQTLs from a subset of GTEx tissues—and Alonso *et al.* (2021)—colocalization of T2D GWAS data with islets of Langerhans eQTLs (Alonso *et al.*, 2021; Gloudemans *et al.*, 2022). We found that our results replicate 24 of 46 genes from Gloudemans *et al.* (2022), including *PLEKHA1*, *AP3S2*, *HMG20A*, and 16 of the 31 genes from Alonso *et al.* (2021), including *HMBS*, *PCBD1*, and *USP36* (Alonso *et al.*, 2021; Gloudemans *et al.*, 2022).

## 4 Discussion

There are a number of ways ColocQuiaL could be used for colocalization analyses that we have not explicitly discussed here. One that we would like to point out to users interested in multi-trait GWAS is that a user can simply run the pipeline once for each trait in a multi-trait analysis in order to assess the evidence that the traits share a causal QTL variant at a locus. There are also a number of other features we plan to add to the ColocQuiaL software over time, including compatibility with other QTL data types and the use of other colocalization methods, such as COLOC-SuSiE to account for loci with multiple causal variants and HyPrColoc to allow for rapid colocalization of three or more traits at a locus (Foley *et al.*, 2021; Wallace, 2021). In summary, ColocQuiaL provides a scalable framework to perform colocalization analyses across the genome between an arbitrary GWAS of interest and any eQTL/sQTL datasets for which a user has summary statistics available. It returns user-friendly summary files and regional association plots for reviewing of the results, allowing users to efficiently generate causal gene and tissue hypotheses for their GWAS results.

## Funding

This work was supported by the American Heart Association [20PRE35120109 to W.P.B.] and National Institutes of Health [DK101478 and DK126194 to B.F.V.].

*Conflict of Interest*: M.D.R. is on the scientific advisory board for Goldfinch Bio and Cipherome. The remaining authors declare no conflicts of interest.

## Data Availability

The T2D summary data utilized in this work are available from dbGAP under accession number phs001672. GT Ex data used for this work are available at their home portal (https://www.gtexportal.org). Predicted causal gene list for T2D was accessed at the T2D knowledge portal (https://t2d.hugeamp.org/phenotype.html?phenotype=T2D), accessed October 1st, 2021.
